# LEP G2548A polymorphism is associated with increased serum leptin and insulin resistance among T2DM Malaysian patients

**DOI:** 10.37796/2211-8039.1326

**Published:** 2022-09-01

**Authors:** Layth Ahmed Ali, Khairunadwa Jemon, Nurriza Ab Latif, Suhaili Abu Bakar, Sharifah Sakinah Syed Alwi

**Affiliations:** a Department of Biosciences, Faculty of Science, Universiti Teknologi Malaysia, 81310, Skudai, Johor, Malaysia; b Department of Biomedical Science, Faculty of Medicine and Health Sciences, Universiti Putra Malaysia, 43400, Selangor, Malaysia

**Keywords:** Leptin, insulin, G2548A polymorphism, type 2 diabetes mellitus, Malaysian's population

## Abstract

**Background:**

Type 2 diabetes mellitus (T2DM) is a chronic metabolic syndrome that is rapidly increasing across the world, especially in Malaysia. Leptin plays a vital role in the regulation of metabolism through its effect on peripheral tissues. G2548A polymorphism in the LEP gene promoter has been associated with insulin resistance, leptin, and type 2 diabetes mellitus across different population, but has not been inclusively reported within the Malaysian population.

**Objective:**

Thus, our study aimed to investigate the impact of G2548A polymorphism on serum leptin levels and insulin resistance among Malaysian T2DM patients.

**Methods:**

This case-control study involved 150 T2DM patients and 150 non-diabetic volunteers from ethnic Malays, Chinese and Indians. Genotyping of G2548A polymorphism was carried out using PCR-RFLP. Serum leptin and insulin levels were determined via ELISA. ANOVA and Chi-square tests were used to determine the distribution of genotypes and allelic frequencies based on serum leptin and insulin levels.

**Results:**

Frequency of AA genotype and A allele of G2548A variant were significantly (P < 0.05) higher in T2DM patients of Malay and Indian ethnicities (4%, 35%, and 36%, 57%, respectively) as compared to the control groups (0%, 22%, and 18%, 35%, respectively). Fasting serum leptin levels were significantly (P < 0.001) higher in T2DM patients compared to non-diabetic subjects (166.78 pg/ml, 101.94 pg/ml, respectively). Additionally, elevated serum leptin, insulin levels, and BMI in diabetic patients were found to be associated with the AA genotype of this variant, compared to GG, and GA genotypes (P < 0.05).

**Conclusion:**

Our findings suggest a significant association between G2548A polymorphism among Malaysian T2DM subjects, particularly among Malay and Indian ethnic groups. Moreover, the A allele frequency of the G2548A variant significantly increased the risk of T2DM and is significantly associated with increased serum leptin, insulin levels, and elevated BMI.

## 1. Introduction

Type 2 diabetes mellitus (T2DM) is a major public health issue worldwide. It is a result of impaired insulin secretion or insulin resistance and is characterized by hyperglycemia [1]. The prevalence of diabetes has significantly risen in recent decades. In 2019, around 463 million patients suffered from diabetes, affecting about 9.3% of the global adult population. This number is predicted to increase to almost 578 million individuals by 2030 [2]. T2DM among Malaysians have become more prevalent in recent years. There are three major ethnic groups in Malaysia, all with differing occurrences of diabetes. The highest incidences were observed among the Indian ethnic group, followed by Malays, and Chinese (28%, 20%, 15%, respectively) [3]. T2DM is a heterogeneous and polygenic disease strongly associated with both macrovascular and microvascular complications such as cardiovascular disease, and diabetic retinopathy [4].

On the other hand, multiple genetic and environmental factors and their interactions have shown to play significant roles in the development of T2DM [5]. Several previous studies have shown obesity to have a significant positive correlation with T2DM and insulin resistance [5–7]. Adipose tissue is an endocrine organ that acts as a central, metabolically dynamic organ in the body [8]. It plays an important role in the regulation of carbohydrate metabolism, energy homeostasis and is the predominant site for storage of excess energy [9]. These actions are triggered by several hormones released by the adipose tissue, known as adipokines [8,9]. One of the most important hormones that regulates energy metabolism and food intake is leptin (*LEP*). Leptin is a peptide hormone mainly released by adipose tissue, and is often referred to as adipocytokine [10]. It plays a crucial role in controlling appetite and energy expenditure through interactions with its receptor that is expressed in the hypothalamus [11]. In humans, leptin levels are highly dependent on the presence of fat in the adipose tissue [11].

Under normal conditions, leptin is able to reduce food intake and increase energy consumption. Leptin is also notably known for its ability to facilitate glucose utilization and improve insulin sensitivity within peripheral tissues [12]. Several studies have reported hyperleptinemia and leptin resistance in humans to be risk factors for various diseases such as obesity, high blood pressure, cardiovascular disease, insulin resistance, and T2DM [13–15]. Leptin resistance decreases the ability of leptin to regulate energy expenditure and appetite, resulting in weight gain as seen in many cases [16,17]. There are many factors that lead to leptin resistance such as leptin signaling defects, lipotoxicity, and inflammatory reactions, which then leads to impaired transmission of messages within cells [18].

Apart from genetic factors, human leptin is encoded by the leptin gene on chromosome 7q32.1 [19]. It is highly polymorphic, and a number of single-nucleotide polymorphisms (SNPs) have been found in promoters, exons, and introns regions of this gene [20]. Several studies have reported the associations of human leptin gene with obesity, insulin resistance, and diabetes mellitus across different population [19,21,22]. From which, leptin promoter G2548A (rs7799039: guanine > adenine) polymorphism was reported to be associated with increased leptin level, obesity, breast cancer, insulin resistance, and T2DM in many populations [23–26]. However, the *LEP* G2548A gene polymorphism among Malaysian diabetes patients has not been investigated yet. Therefore, this study investigated the effect of LEP G2548A on leptin levels and insulin resistance in T2DM among Malaysian subjects.

## 2. Subjects and methods

### 2.1. Study ethics

The study's protocol has been registered under the National Medical Research Registry (NMRR-19-1242-46808) and ethics approval was obtained from the Medical Research and Ethics Committee (MREC), Ministry of Health Malaysia (MOH) and Clinical Research Centre (CRC), Hospital Serdang. All participants involved in this study were volunteers and had signed the informed consent forms prior to sample collection.

### 2.2. Study population

A case control study was conducted among 300 subjects at Hospital Serdang, Selangor between September 2019 and December 2020. Patient selection was done in accordance with necessary inclusion and exclusion criteria. The practical segment of the study was performed at the Department of Biomedical Science, Faculty of Medicine and Health Sciences, University Putra Malaysia. A questionnaire assessing demographic data such as age, weight, height, body mass index, ethnicity, duration of illness, family history of T2DM, medications, and blood pressure was administered in both Malay and English languages.

#### 2.2.1. Inclusion and exclusion criteria

A total of 150 cases with confirmed T2DM (≥ 18 years) across the three major ethnic groups in Malaysia (i.e. 50 Malays, 50 Chinese, and 50 Indians) were recruited and selected according to criteria set by the International Diabetes Federation (IDF). Any acute and chronic diseases other than T2DM such as T1DM, cardiovascular diseases, hepatitis diseases, nephropathy, hypertension, pregnancy among others were excluded from the study.

A group of 150 non-diabetic individuals from the three ethnic groups was also included in the study as a control group. Participants were randomly selected from those who visited the hospital for medical examinations, their weight, gender, and race comparable to that of enrolled patients. Moreover, none of these subjects had any obvious systemic disease.

### 2.3. Blood sample collection

Fasting venous blood samples (4 ml) from diabetic patients and healthy individuals were withdrawn into EDTA tubes to avoid blood clotting. After that, one ml of human blood sample was used for DNA extraction, while the remaining were centrifuged at 10000 rpm for 10 min. The resulting serum was then stored at −20 °C to determine the blood leptin and insulin levels.

### 2.4. Phenotypic analysis

Body mass index was calculated (i.e., weight in kg divided by height in m^2^). Enzyme-linked immunosorbent assay (ELISA) kits (Human Leptin and Insulin ELISA kits, Elabscience, China) were used to estimate the concentration of fasting serum insulin and leptin, based on manufacturer instructions. Fasting blood glucose level was measured by glucose oxidase method. Insulin resistance was evaluated by the homeostasis model assessment of insulin resistance (HOMA-IR), calculated based on the formula: HOMA-IR = [Fasting insulin (μIU/ ml) × Fasting glucose (mmol/l)] /22.5 [27].

### 2.5. DNA extraction from whole blood

Genomic DNA was extracted from the whole blood sample using QIAamp DNA Mini Kit (Qiagen, Germany) in accordance with the QIAamp procedure. The quality of the DNA samples was assessed via electrophoresis on 2% agarose gel and the quantity of extracted DNA was evaluated via Nanodrop method. Purity of the DNA samples was determined by calculating the ratio of absorbance at 260 nm to the absorbance at 280 nm (A260 / A280). Pure DNA samples should have an absorbance ratio of 260/280 from 1.7 to 1.9 - any value beyond this range indicates that the sample is contaminated with proteins and other contaminants.

### 2.6. Genotyping analysis

Genotyping of LEP G2548A polymorphism was detected by polymerase chain reaction-restriction fragment length polymorphism (PCR-RFLP) method. The forward primer sequence was 5’-TAA GCC AAG GCA AAA TTG AG-3’ and reverse primer sequence was 5’-CTT CAA AAT TTA TGT TCC TCT GC-3’. PCR amplification was performed in a 15 μl PCR tube per sample, each consisting of 7.5 μl of the master mix, 6.1 μl of ultra-pure water, 0.2 μl forward and reverse primers respectively, and 1 μl genomic DNA.

The PCR protocol was carried out in a thermo-cycler machine, with hot start at (95 °C, 2 min), followed by denaturation at (95 °C, 1 min), annealing at (57 °C, 1 min), and extension at (72 °C, 1 min) in 35 cycles with a final extension at (72 °C, 10 min). The resultant PCR product was then verified by electrophoresis on 2% agarose gel. Next, the PCR product of 281 bp was digested with *HhaI* restriction enzyme (New England Biolabs, USA) at 37 °C for 20 min and then inactivated for 20 min at 65 °C. Finally, the RFLP product size was performed on 3% agarose gel and the electrophoresis was performed at 50 V for 45 min, before it was viewed under UV light.

### 2.7. Data validation

The genotyping results of leptin gene polymorphism were performed twice by selecting 10% of randomized samples to verify accuracy of the results. It was 100% accurate when compared to previous results.

### 2.8. Statistical analysis

Statistical analyses were carried out via Statistical Package for Social Sciences (SPSS Inc. Chicago, IL, USA, version 17.0), with level of significance at *P* ≤ 0.05. Results were expressed in means and standard deviations. T-test was administered to observe differences in demographic and clinical characteristics between T2DM cases and control groups. ANOVA was used to compare average values of biochemical parameters between genotypes. Allelic frequencies were estimated by gene counting. Chi-square (χ2) test was used to determine the distribution of genotypes and allelic frequencies between groups. Pearson's correlation was used to determine the correlation of age, leptin, and insulin with clinical and biochemical parameters among T2DM patients. Odds ratio with a 95% confidence interval was also calculated.

## 3. Results

### 3.1. Baseline clinical and biochemical parameters of the study groups

Baseline characteristics of the study population (T2DM patients and non-diabetic subjects) are as summarized in Table 1. The average age, fasting blood sugar, fasting serum insulin, HOMA-IR, and serum leptin level were significantly higher in T2DM patients compared to non-diabetic subjects (P > 0.001). Additionally, family history of T2DM was more frequent in the case group than in the control group. However, no significant differences (P < 0.05) in weight, height, BMI, gender, systolic and diastolic blood pressure were observed between these groups.

Logistic regression analysis was used to evaluate the correlation of age, leptin, and insulin with clinical and biochemical parameters among T2DM patients as presented in Table 2. The results demonstrated the significant negative correlation between the age of T2DM patients with BMI, serum insulin, and serum leptin (P < 0.05) and positive correlation with SBP and DBP (P < 0.05). However, no significant correlation was observed between age with FBS and HOMA-IR among T2DM patients. On the other hand, serum leptin and insulin levels were significantly positive correlated with BMI, and HOMA-IR (P < 0.05), but negatively correlated with SBP, DBP. Strong positive correlation was observed between leptin and insulin levels in T2DM patients (P < 0.001). However, no significant negative correlation of leptin and insulin were observed with FBS (P < 0.05).

### 3.2. Distribution of genotypic and allelic frequencies of G2548A variant

**Fig. 1:**
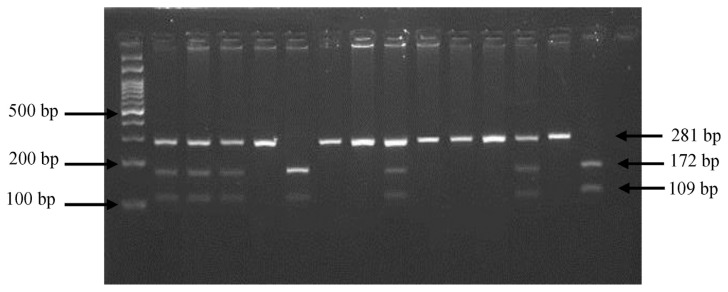
The enzymatic digestion of G2548A on 3% agarose gel. L is a 100 bp DNA ladder. GA (281, 172, 109 bp) indicates heterozygous. AA (172, 109 pb) indicates homozygous. GG (281 bp) indicates wild type.

As shown in Fig. 1, this study detected three bands of the G2548A polymorphism once the PCR product was digested with *HhaI* restriction enzyme; 281 bp for GG, 172 bp and 109 bp for AA, 281 bp, 172 bp, and 109 bp for GA. The genotype and allele frequencies of G2548A polymorphism for both the case and control groups is as tabulated in Table 3. There was a significant difference in the genotypic model and allelic frequencies of LEP promoter gene (G2548A) polymorphism between T2DM and non-diabetes subjects (P > 0.05).

The GG genotype and G allele were the most common genotypic and allelic frequencies for SNP of G2548A among T2DM and control groups. The frequency of GG genotype was less frequent among T2DM patients (34%) than the non-diabetic individuals (53.34%). However, the AA and GA genotypes distribution was significantly higher in T2DM patients (16.66%, 49.34%, respectively) than the non-diabetic group (χ2 = 13.55, P = 0.001). Furthermore, the allele frequencies G and A of LEP G2548A among T2DM (58.66%, 41.34%, respectively) were significantly different (χ2 = 13.7, P < 0.001) from non-diabetic subjects (G = 73%, A = 27%).

Table 4 demonstrates the genotype and allele frequencies of G2548A across the three different ethnicities. There were significant differences in genotypic and allelic frequencies were observed between T2DM and control groups for the Malay and Indian ethnic groups (P = 0.045, P = 0.016, respectively). While no significant differences were observed among Chinese subjects (p = 0.392). The odds ratio of dominant genetic model for Malays were 2.47 (95% CI: 1.1–5.5, P = 0.027). Whereas dominant genetic model for Indian ethnic revealed odds ratio of 3.27 (95% CI: 1.37–7.81, P = 0.006), with recessive model conferring 2.56-fold risk towards T2DM development (OR: 2.56, 95% CI: 1.02–6.46, P = 0.043). Among the Malay ethnic group, frequency of A allele was significantly higher in T2DM patients than non-diabetic subjects (0.35 vs 0.22, OR = 1.9 (1.02–3.57), P = 0.042). While A allele frequency was observed to be 0.57 in Indians with T2DM compared to 0.35 in non-diabetic Indians (OR = 2.46 (1.39–4.36), P = 0.002).

The relationship between SNP LEP G2548A genotypes with clinical and biochemical parameters in T2DM patients and control groups is presented in Table 5. The results showed that T2DM patients with AA genotype had significantly higher BMI, serum insulin, and serum leptin levels (P < 0.05) compared to those with GG and GA genotypes. Nevertheless, there were no significant associations between LEP G2548A variant with age, SBP, DBP, FBS, and HOMA-IR index (P < 0.05) in T2DM patients. No significant association was observed between LEP G2548A polymorphism with clinical and biochemical parameters in control group (P < 0.05).

## 4. Discussion

To our knowledge, this is the first study that determines the association between G2548A variant in *LEP* gene with T2DM among the Malaysian population. The main findings of this study were that *LEP* G2548A polymorphism was significantly associated with T2DM among the Malaysian population, and correlated with BMI, serum leptin, and insulin levels in T2DM patients. In addition, there was a significant elevation of serum leptin levels in T2DM patients in comparison to the non-diabetic control group. Several studies have reported that hyperleptinemia is associated with insulin resistance and T2DM across different populations [14,15,28,29].

The peripheral action of leptin is to inhibit insulin secretion and biosynthesis in pancreatic β cells through receptors present in beta cells of pancreas. Insulin on the other hand stimulates secretion of leptin from adipose tissue. The relationship between pancreas and adipocytes is referred to as “adipo-insular axis”, which plays as key role in modulation of body metabolism [30]. Therefore, dysregulation of the adipo-insular axis could be a result of leptin resistance in the pancreatic cells, hence contributing to the development of insulin resistance and type 2 diabetes.

Our findings revealed that the average age of T2DM patients was significantly higher than non-diabetic participants (P < 0.001) as summarized in Table 1. This suggests that the elderly age group has an increased risk of developing T2DM. Moreover, the age was significantly negative correlated with BMI (r = − 0.318, P < 0.001), fasting insulin (r = − 0.258, P = 0.001), and serum leptin (r = − 0.221, P = 0.007) and positively correlated with SBP, and DBP (r = 0.772, r = 0.738, P < 0.001) as presented in Table 2. This association is in line with previous studies [31–33].

Our study also found that fasting serum leptin concentrations was positively correlated with BMI among T2DM patients (r = 0.902, P < 0.001). This leptin-obesity relationship has been reported in a multi-ethnic population as well as in T2DM patients [32,34–36]. Furthermore, leptin levels were found to be positively correlated with fasting serum insulin concentrations and HOMA-IR (r = 0.603, r = 0.509, P < 0.001, respectively) as shown in Table 2. These findings are in accordance with data obtained from previous studies in T2DM patients [35,36]. Reduced leptin signaling in pancreatic beta cells due to leptin resistance leads to increased secretion of insulin (hyperinsulinemia) [37]. Chronic hypersecretion of insulin promotes both insulin resistance and hypersecretion of leptin from the adipose tissue [30]. Additionally, the lack of tonic suppression of insulin secretion by leptin, leads to increased insulin production by pancreatic beta cells, which then results in pancreatic beta cell dysfunction – a major cause of type 2 diabetes mellitus [38]. Failure of insulin to uptake glucose into target tissues such as muscle, liver, and adipose due to insulin resistance will lead to hyperglycemia [39].

Findings from our study as presented in Table 3 demonstrated that the genotypic and allelic frequencies of polymorphism G2548A of LEP gene were significantly different (P > 0.05) between T2DM patients and non-diabetic control group. T2DM patients had significantly higher AA genotype and A allele frequency of LEP gene polymorphism than non-diabetic group. Conversely, the frequency of G allele and GG genotype distribution were significantly lower in T2DM patients than non-diabetic control group. This is in accordance with data obtained from previous studies. A study performed by Meshkani et al. reported that the AA genotype of G2548A variant and A allele frequency were significantly higher in patients with T2DM than in normoglycemis subjects [40]. Another study by Roszkowska-Gancarz et al. found that leptin gene promoter polymorphism was associated with diabetic patients [41]. In addition, Bains et al. observed that leptin gene G2548A polymorphism demonstrated significant associations with T2DM patients [42]. In contrast to our results, some studies showed no difference in genotypic and allelic frequencies of leptin gene promoter G2548A variant between diabetic patients and non-diabetic subjects [29,43,44]. Conflicting results may be due to differences in races, genetic backgrounds of the study population, sample size of the population, and geographic variation.

Malaysia is a multi-ethnic country with ethnic majority comprising of Malays, followed by Chinese, and Indians. The present study also investigated the distribution of genotypic and allelic frequencies of LEP gene polymorphism G2548A across these three major ethnic groups as shown in Table 4. The proportion of Malay, Chinese, and Indian was matched between the two groups. Among the Malay ethnic group, our study observed significant differences in genotypic and allelic frequencies of leptin gene polymorphism of G2548A in T2DM subjects as compared to control subjects (P > 0.05). From which approximately, 34% had GG genotype, 62% had GA genotype, and only 4% had AA genotypes as compared to normoglycemic Malay subjects, where 56%, 44%, and 0% had GG, GA, and AA genotypes, respectively. Besides that, the A allele frequency was significantly higher in Malay T2DM than its control group (0.35, and 0.22, respectively). On the other hand, there was no significant differences between Chinese T2DM and control group in genotypic and allelic frequencies of G2548A polymorphism (P < 0.05). This is in agreement with results from a study conducted by Yang et al. (2016), where no links were found between LEP gene G2548A polymorphism and T2DM within the Chinese population [29].

The present study also demonstrated distribution of genotypes and allelic frequencies of variant G2548A among the Indian ethnic group. There were significant differences (P > 0.05) in genotypic and allelic frequencies between the two groups as presented in Table 4. T2DM Indians patients had 22% GG genotype, 42% GA, and 36% AA genotypes, as compared to its control group where the frequency of GG, GA, and AA genotypes were 48%, 34%, and 18%, respectively. In addition, the frequency of A allele of G2548A polymorphism was higher in Indians with T2DM than non-diabetic individuals (0.57, and 0.35, respectively). In other words, the A allele frequency of this variant was significantly associated with T2DM among the Indian ethnic group, similar to that by Meshkani et al. (2016) who reported lower G allele frequency of G2548A polymorphism than A allele frequency in T2DM patients [40]. Moreover, Kohan et al. (2013) found that the LEP gene polymorphism of G2548A increased the risk of T2DM [45].

The relationship between SNP of G2548A in the LEP gene promoter and the demographic risk factor of the study population was also evaluated in our study. From this study, it was observed that carriers of the AA genotype of LEP gene G2548A variant had significantly higher BMI compared to carriers of the GG genotypes among T2DM patients as summarized in Table 5. Our findings were in line with other studies like that by Boumaiza et al. (2012) who found higher BMI in AA genotype of leptin gene polymorphism than GG genotype among Tunisian subjects [46]. Likewise, studies conducted among Mexican, Turkish, and Indian obese patients have reported association between G2548A variants with higher BMI in subjects with AA genotype, excluding GG and GA genotypes [47–49].

In our observation, serum leptin and insulin levels of T2DM patients were significantly higher in those AA genotype in comparison to the other genotypes as stated in Table 5. Similarly, previous studies have shown that the AA genotype of SNP G2548A was associated with increased leptin levels, suggesting that the G2548A variant in the leptin gene may stimulate leptin resistance [48,50]. A recent study suggested that the variation of G2548A in the promoter of the leptin gene may affect leptin gene expression and leptin secretion by adipose tissue [23]. Additionally, a study found that Tunisian subjects with AA genotype were shown to have higher fasting insulin concentration compared to those with GG genotype [46]. The limitation of our study is the small sample size. Therefore, a larger sample size comprising different Malaysian ethnic groups and subjects from all Malaysian states is required for future studies to confirm current findings.

## 5. Conclusion

The present study revealed that G2548A (rs7799039) polymorphism of the LEP gene was strongly associated with T2DM within the Malaysian population, especially among Malay and Indian ethnic groups. The AA genotype of this variant was significantly associated with increased BMI, fasting leptin, and insulin levels in patients with T2DM. In addition, high serum leptin concentration was significantly correlated with T2DM patients. Our findings could be a useful genetic marker for identifying patients with T2DM and may serve as basis for future research and investigation.

## Figures and Tables

**Table 1:** Clinical and biochemical characteristics of the study population.

Variables	Population		P-value

	T2DM (n = 150)	Non-diabetic (n = 150)	
Age (years)	58.16 ± 11.07	44.75 ± 13.2	<0.001*
Gender:			
Male	88 (58.66%)	85 (56.66%)	0.726
Female	62 (41.34%)	65 (43.34%)	0.260
Weight (kg)	70.78 ± 11.37	72.18 ± 9.94	0.283
Height (cm)	1.64 ± 0.07	1.65 ± 0.07	0.388
BMI (kg/m^2^)	26.11 ± 2.96	26.38 ± 2.55	0.095
SBP (mm Hg)	117.10 ± 4.07	116.27 ± 4.53	0.074
DBP (mm Hg)	75.47 ± 3.72	74.63 ± 4.30	<0.001*
FBS (mmol/L)	10.99 ± 2.71	4.83 ± 0.47	<0.001*
Insulin (μIU/ml)	19.83 ± 8.88	11.24 ± 5.58	<0.001*
HOMA-IR	9.60 ± 5.06	2.43 ± 1.29	<0.001*
Leptin (Pg/ml)	166.78 ± 60.05	101.94 ± 45.04	
Family History			
Yes	69 (46%)	51 (34%)	0.034*
No	81 (54%)	99 (66%)	
Smoking			
Yes	52 (34.36%)	62 (41.34%)	0.234
No	98 (65.34%)	88 (58.66%)	

Continuous variables were presented as mean ± standard deviation (SD) and compared by independent t-test. Categorical variables were compared by χ2.

* P ≤ 0.05 was considered as significant value.

BMI, body mass index; SBP, systolic blood pressure; DBP, diastolic blood pressure; FBG, fasting blood sugar.

**Table 2:** The Correlations of age, leptin, and insulin with clinical and biochemical parameters in T2DM patients.

Parameters	Age		Leptin		Insulin	
		
	r	P-value	r	P-value	R	P- value
BMI (kg/m^2^)	−0.318	< 0.001	0.902	< 0.001	0.674	< 0.001
SBP (mm Hg)	0.772	< 0.001	−0.188	0.021	−0.292	< 0.001
DBP (mm Hg)	0.738	< 0.001	−0.216	0.008	−0.286	< 0.001
FBS (mmol/L)	0.167	0.141	−0.031	0.702	−0.079	0.339
Insulin (μIU/ml)	−0.258	0.001	0.603	< 0.001	—	–‘
Leptin (Pg/ml)	−0.221	0.007	—	—	0.603	< 0.001
HOMA-IR	−0.158	0.054	0.509	< 0.001	0.824	< 0.001

r, Pearson's correlation coefficient; BMI, body mass index; SBP, systolic blood pressure; DBP, diastolic blood pressure; FBG, fasting blood sugar.

**Table 3:** Genotype and allele frequency of LEP G2548A polymorphism in T2DM and non-diabetic individuals among Malaysian subjects.

Model	Genotype/Allele	*LEP (G2548A)*		χ^2^ value	P-value

		T2DM (n = 150) No. (%)	Non-diabetic (n = 150) No. (%)		
Codominant	GG	51 (34%)	80 (53.34%)	13.55	0.001*
	GA	74 (49.34%)	59 (39.33%)		
	AA	25 (16.66%)	11 (7.33%)		
Dominant	GG	51 (34%)	80 (53.34%)	11.4	0.001*
	GA + AA	99 (66%)	70 (46.66%)		
Recessive	AA	25 (16.66%)	11 (7.34)	6.187	0.013*
	GA + GG	125 (83.34%)	139 (92.66)		
Allele	G	176 (58.66%)	219 (73%)	13.7	<0.001*
	A	124 (41.34%)	81 (27%)		

Genotypes and allele frequencies were compared using Chi-square.

* P ≤ 0.05 was considered as statistically significant value.

**Table 4:** Genotype and allele frequencies of LEP G2548A across the three ethnicities.

Ethnicity	Genotype/Allele	T2DM (n = 50) No. (%)	Non-diabetic (n = 50) No. (%)	P-value	OR (95% CI)
Malay (n = 100)	GG	17 (34%)	28 (56%)	0.045*	–
	GA	31 (62%)	22 (44%)		
	AA	2 (4%)	0 (0%)		
	GG	17 (34%)	28 (56%)	0.027*	2.47 (1.1, 5.5)
	GA + AA	33 (66%)	22 (44%)		
	AA	2 (4%)	0 (0%)	0.153	–
	GA + GG	48 (96%)	50 (100)		
	G	65 (65%)	78 (78%)	0.042*	1.9 (1.02, 3.57)
	A	35 (35%)	22 (22%)		
Chinese (n = 100)	GG	23 (46%)	28 (56%)	0.392	2.27 (0.4, 13.05)
	GA	22 (44%)	20 (40%)		
	AA	5 (10%)	2 (4%)		
	GG	23 (46%)	28 (56%)	0.317	1.49 (0.68, 3.29)
	GA + AA	27 (54%)	22 (44%)		
	AA	5 (10%)	2 (4%)	0.240	2.67 (0.5, 14.44)
	GA + GG	45 (90%)	48 (96%)		
	G	68 (68%)	76 (76%)	0.208	1.5 (0.8, 2.78)
	A	32 (32%)	24 (24%)		
Indian (n = 100)	GG	11 (22%)	24 (48%)	0.016*	1.62 (0.58, 4.5)
	GA	21 (42%)	17 (34%)		
	AA	18 (36%)	9 (18%)		
	GG	11 (22%)	24 (48%)	0.006*	3.27 (1.37, 7.81)
	GA + AA	39 (78%)	26 (52%)		
	AA	18 (36%)	9 (18%)	0.043*	2.56 (1.02, 6.46)
	GA + GG	32 (64%)	41 (82%)		
	G	43 (43%)	65 (65%)	0.002*	2.46 (1.39, 4.36)
	A	57 (57%)	35 (35%)		

Genotypes and allele frequencies were compared using Chi-square.

* P ≤ 0.05 was considered as statistically significant value.

OR: odd ratio. CI: confidence interval.

**Table 5:** Association of LEP G2548A polymorphism with anthropometric parameters in T2DM patients and control groups.

Variables	T2DM			P- value	Control			P- value
	
	GG	GA	AA		GG	GA	AA	
Age (years)	59.35 ± 12.43	56.85 ± 10.66	59.60 ± 8.93	0.361	45.34 ± 12.84	43.86 ± 13.73	45.27 ± 13.78	0.804
BMI (kg/m^2^)	25.53 ± 2.17	26.25 ± 2.73	27.35 ± 3.51	0.024*	26.25 ± 2.6	26.45 ± 2.6	26.92 ± 2.03	0.693
SBP (mm Hg)	117.35 ± 4.5	116.42 ± 3.84	118.60 ± 3.39	0.058	116.12 ± 4.43	116.44 ± 4.55	116.36 ± 5.52	0.916
DBP (mm Hg)	75.19 ± 3.73	75.13 ± 3.60	77.00 ± 3.82	0.078	74.68 ± 3.92	74.57 ± 4.57	74.55 ± 5.68	0.986
FBS (mmol/l)	11.50 ± 2.94	10.69 ± 2.70	10.82 ± 2.1	0.248	4.87 ± 0.5	4.78 ± 0.44	4.76 ± 0.36	0.487
Insulin (μIU/ml)	18.22 ± 7.76	19.56 ± 9.17	23.93 ± 9.2	0.028*	11.14 ± 5.41	11.18 ± 6.07	12.23 ± 4.20	0.831
HOMA-IR	9.17 ± 4.55	9.26 ± 5.34	11.48 ± 4.96	0.125	2.42 ± 1.25	2.40 ± 1.40	2.60 ± 0.98	0.894
Leptin (pg/ml)	153.74 ± 46.3	167.00 ± 58.45	192.7 ± 80.4	0.028*	101.30 ± 44.72	102.20 ± 48.28	105.18 ± 29.92	0.964

The results were presented as mean ± standard deviation (SD). One-way analysis of variance (ANOVA) was used to determine the different between genotypes of the polymorphism.

* P ≤ 0.05 was considered as statistically significant value with 95% confidence intervals.

BMI, body mass index; SBP, systolic blood pressure; DBP, diastolic blood pressure; FBG, fasting blood sugar.
